# Capsule endoscopy in airway: a difficult extraction

**DOI:** 10.1093/jscr/rjac385

**Published:** 2022-08-30

**Authors:** Sandeep Tamang, Sergei Mitnovetski

**Affiliations:** Thoracic Department, Northern Health Melbourne, Victoria, Australia; Thoracic Department, Northern Health Melbourne, Victoria, Australia

## Abstract

Foreign bodies of different nature are identified in airway. Extraction of foreign bodies given their dimension and character requires proper set of instruments with right technique to ensure safe removal. Different tools have been discussed to optimally withdraw the object in various articles which have been applied. However, we talk about a case of removal of a pill camera from an elderly patient’s right main bronchus with a certain positioning of the patient after multiple unsuccessful attempts with instruments alone. In conclusion, pill camera as being useful for surveillance for gastrointestinal pathology can have the potential of aspiration.

## INTRODUCTION

Foreign body aspiration is an uncommon but potentially life-threatening problem. Commonly aspirated objects include a variety of inanimate objects. However, they can also be live, e.g. leeches and roundworms [1]. Video capsule endoscopy, which uses a wireless camera, has been used since 2001, aids in the diagnosis and management of many pathologies of the small and large bowel [2]. Although thought to be safe and easy for the patient, aspiration of the capsule has been an increasingly reported complication, especially in the older age group [3]. Emergent management of such an accident requires prompt identification and adequate expertise. Herein, we report a case of an aspirated capsule managed successfully endoscopically.

## CASE REPORT

**Figure 1:**
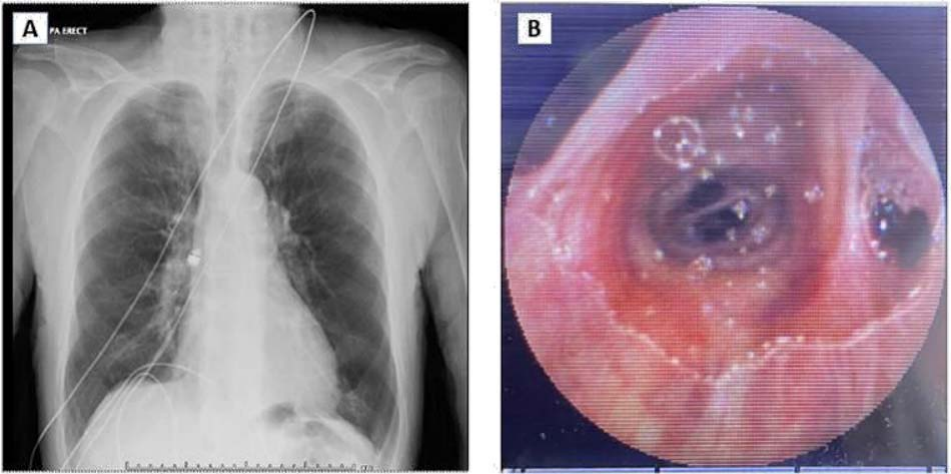
(**A**) Chest X-ray showing capsule in the right main bronchus. (**B**) Capsule lodged in bronchus intermedius showing endobronchial anatomy.

An 87-year-old gentleman was investigated for anaemia, and a positive faecal blood test was booked for capsule endoscopy. This was done as an outpatient procedure using a Pillcam SB 3 system (Medtronic, MN, USA). After necessary directions, the patient was asked to swallow the capsule. Following swallowing of the capsule, the patient started to cough intermittently and desaturated requiring supplemental oxygen. On the monitor, the capsule was not moving and did not appear to be in the oesophagus. Chest X-ray confirmed the pill was in the right main bronchus (Fig. 1A). The patient was then referred to the Thoracic Surgical service for urgent evaluation and management.

**Figure 2:**
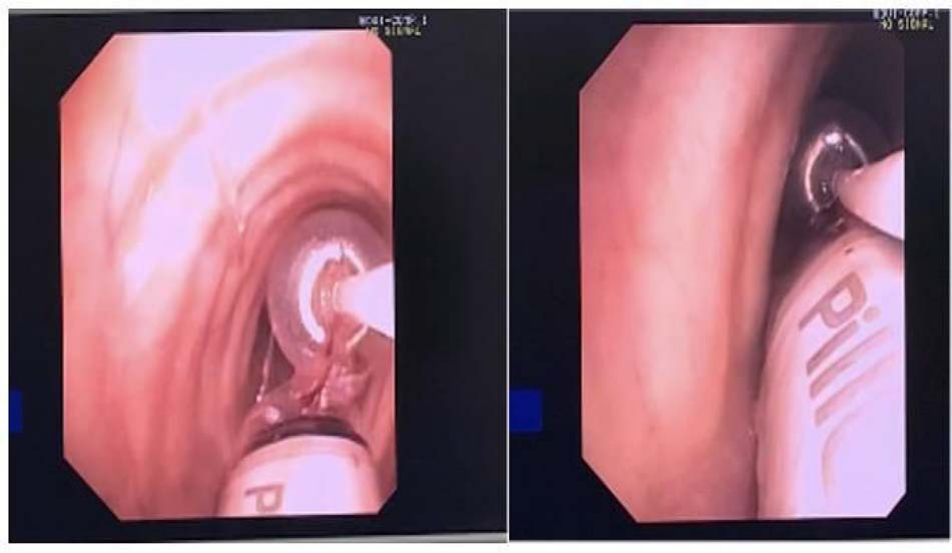
Fogarty balloon Catheter showing removal of Pill Cam.

Post review, the patient consented to rigid/flexible bronchoscopy and retrieval of a foreign body. Because of the prevalent coronavirus disease 2019 (COVID-19) pandemic, the procedure was performed with full COVID precautions. A flexible bronchoscope evaluation was performed with the patient under general anaesthesia and through a size 8.5 endotracheal tube (ETT). The capsule was found to be lodged in the bronchus intermedius (Fig. 1B). The size of the capsule (26.2 × 11.4 mm) and its very smooth surface meant that it could not be grasped by available endobronchial instruments. Attempts to trap it in a Nitnol tipless stone extractor basket (2.2 cm) were not successful. We subsequently tried using Fogarty catheters (of sizes 3–7). Although we were able to dislodge the capsule into the trachea, the size discrepancy between the balloon and trachea and perhaps because of the weight of the capsule, it could not be delivered into the upper trachea it repeatedly slipped back to the bronchus on the right. Because of this repeated slippage, the patient was placed in a stip Trendelenburg position. The use of gravity prevented slippage of the capsule distally. After this manoeuvre, the capsule was drawn to the tip of the ETT. The capsule was wedged between the balloon of the Fogarty and the ETT tip and they were all withdrawn as a unit (Fig. 2). The capsule was brought outside the vocal cords and retrieved using Magill forceps. The patient was subsequently reintubated until subsequent extubation. The postoperative recovery was uneventful.

## DISCUSSION

An advertent aspiration is a known complication of capsule endoscopy occurring about 1/1000 cases [4] and has the potential to lead to a significant clinical problem especially in the elderly [5]. The size and the smoothness of the capsule makes extraction difficult and conventional grasping forceps are not usually successful. A variety of endobronchial tools have been used including snares [4], Roth Net retrieval device, stone basket, Roth Net expandable foreign body extractor, Fogarty Balloon catheter, rat-toothed graspers through flexible or rigid bronchoscope [6] have been described. In our case, despite the use of available devices, extraction proved difficult. Success was only attained after patient was put in Trendelenburg position and the capsule was wedged between the balloon of the Fogarty and the tip of ETT. This manoeuvre though successful does require help and co-ordination of the entire theatre team to ensure patient remains secure on the operating table.

## CONCLUSION

Despite being a useful tool in the diagnostic armamentarium of the gastroenterologists, capsule endoscopes do have the potential of aspiration. Their extraction requires an expertise and appropriate instrumentation. Extraction can be significantly aided, and repeated migration avoided by placing the patient in a stip Trendelenburg position once it has been disimpacted.
